# Licensing of Generic Medicines: Are There Any Challenges Left? A Pharmaceutical Regulatory Perspective

**DOI:** 10.3797/scipharm.1312-10

**Published:** 2014-05-22

**Authors:** John Joseph Borg, Paolo Tomasi, Luca Pani, George Aislaitner, Michal Pirozynski, Hubert Leufkens, Daniela Melchiorri

**Affiliations:** ^1^ Medicines Authority, 203 Level 3, Rue D’Argens, Gzira, GZR 1368, Malta.; ^2^ Department of Biology, School of Pharmacy, University of Tor Vergata, Rome, Italy.; ^3^ European Medicines Agency, 7 Westferry Circus, London E14 4HB, UK.; ^4^ Italian Medicines Agency, AIFA, Via del Tritone, 181, 00186 Rome, Italy.; ^5^ National Organisation of Medicines (EOF), 284 Messogion STF, 155 62 Athens, Greece.; ^6^ Department of Anaesthesiology and Critical Care Medicine, Postgraduate MedicalSchool, Czerniakowska 231, 00 416 Warsaw, Poland.; ^7^ Medicines Evaluation Board (MEB), Graadt van Roggenweg 500, 3531 AH Utrecht, Netherlands.; ^8^ Department of Physiology and Pharmacology, University of Rome “Sapienza”, Rome, Italy.

**Keywords:** Generics, Therapeutic equivalence, Biocreep, Off-patent medicines, Regulatory science, Safety of generics

## Abstract

When an innovative product (innovator) is not covered anymore by intellectual property rights, cheaper equivalent medicinal products (generic products) may be marketed and used in clinical practice. The regulation of generic products is well-established, and is primarily based on standard rules for quality, therapeutic equivalence requirements (the latter in most instances proven through a bioequivalence study), and safety data for the innovator. The extensive experience from bringing generic products to the market over the last decades allows the conclusion that they are well-accepted and provide a useful alternative option for cost-effective pharmacotherapy. While supporting this conclusion, there are a number of issues to be considered during the assessment of a generic product application. Six scenarios are described in total, from an efficacy and a safety perspective, where potential concerns with the current regulatory standards could arise in the approval of generic products. We also propose solutions to these scenarios in order to foster debate on these issues.

## Introduction

During the lifecycle of a medicine, there is constant change and updating of relevant features and information. The examination of a medicinal product is never finished and continues throughout its lifecycle. When an innovator product is not covered anymore by intellectual property rights, such as a patent/supplementary protection certificate or data protection granted by the marketing authorization (“off-patent” products), competing products containing the same active substance[s] (“generics”) may enter the market and clinical practice [1]. The extensive experience gained by the authorization and marketing of generics over the last decades allows the conclusion that generic medicinal products are well-accepted. As generics are usually cheaper, often substantially, insurers and healthcare payers in general tend to encourage their prescription and substitution in place of the innovator product. Medicinal product regulators (including all EU national regulatory agencies and the US Food and Drug Administration (FDA)) consider generic and branded drugs to be therapeutically equivalent if they are pharmaceutically equivalent and bioequivalent. However, the American Academy of Neurology disagrees and opposes generic substitution of branded antiepileptic drugs without physician and patient approval due to the risk of loss of seizure control [2]. Thus, we asked the question, “could there be a potential concern with the rules used to approve generics?”

The regulation of generic products is well-established, and it is primarily based on standard rules for the determination of quality, safety, and efficacy (in most instances proven through a bioequivalence study) [1, 3]. In the EU, a generic medicinal product is a medicinal product which has the same qualitative and quantitative composition in active substances and the same pharmaceutical form as the reference medicinal product (the innovator), and whose bioequivalence with the reference medicinal product has been demonstrated by appropriate bioavailability studies, thus supporting equivalent efficacy, unless the requirements for a Biowaiver have been satisfactorily fulfilled [3]. In this paper, we discuss potentially clinically relevant scenarios that impact prescription and patient treatment that could arise when generics are approved with the current standard rules [3].

For the sake of clarity, readers should be aware that in a marketing authorisation application of a generic medicinal product, data on the interchangeability of generics with reference products or other products on the market is not part of a dossier submitted to obtain a marketing authorisation. Substitution therapy varies between EU member states and is regulated at a national level by each member state.

## Results

### Efficacy and Safety Challenges

An indication for a medicinal product (product B) still under patent is applied for (this could either be as an initial marketing authorisation or as a variation to the marketing authorisation). The clinical data package supporting the new indication would include a non-inferiority trial, intended to demonstrate that the clinical effect of product B is not inferior to that of an active control (product A) by more than a specified margin [4]. While it may not be feasible to include a (third) placebo arm, for example due to ethical reasons (products to treat cancer, cardiovascular diseases, epilepsy, etc.), nevertheless, a non-inferiority clinical trial may still be acceptable. For example, product B could have benefits such as a more convenient dosing regimen or route of administration (e.g. oral instead of intramuscular), or better tolerability, or a potential small increase in efficacy (which would require a larger, more costly superiority trial to be demonstrated). However, there is a concern that this may not be sufficiently considered during the subsequent evaluation of a generic medicinal product (product C) of product B. As bioequivalence and non-inferiority trials are specific subtypes of equivalence trials, a specific type of bio-creep can arise here. If the bioavailability of product C is in the lower region of the acceptable range, and product B was declared non-inferior, but had slightly less efficacy than that of product A, then we cannot safely assume that product C will have similar efficacy to that of product A. In other words, bioequivalence of C to B would not guarantee non-inferiority of C to A, even if B is non-inferior to A.

An innovative medicinal product is indicated as monotherapy in the treatment of partial-onset seizures or other epilepsy indications. The active substance exhibits non-linear pharmacokinetics, but also has a wide therapeutic index (i.e. the dose producing a clinical effect and the dose producing an adverse event is wide), while the effective dosing range for this product is narrow. As the active substance has a wide therapeutic index, the conventional 90% confidence interval (CI) limits of 80–125% can be applied here; however, they may not be appropriate in this case: if the bioavailability of the generic is close to the lower acceptance limit, a supposedly equivalent dose of the generic could well lie outside the clinically effective range for the specific therapeutic indication. This could potentially translate into a significant risk of a breakout seizure that may have serious consequences depending on what activity the patient is doing at that precise point in time. This mechanism might have been involved in the case reports of the loss of seizure control with what authors reported as “generics” (drugs reported include phenytoin, carbamazepine, valproic acid, lamotrigine, levetiracetam, and topiramate) [5–14].

In the progression of Parkinson’s disease (PD) from early PD through moderate and then advanced PD, it is known that the margin of separation between the apparently safe and effective dose for medicinal products indicated in PD and the dose causing adverse events decreases as PD progresses. Whereby the basic equivalent dose of levodopa / benserazide in early PD would result in a smooth prolonged duration of a clinical response associated with a low likelihood of dyskinesias, in the case of advanced PD, one would tend to observe a short duration of the targeted clinical response “on time” associated with dyskinesias. Again, the conventional bioequivalence limits (80–125%) are considered appropriate for the approval of a generic product intended to be used during the initial phases of the disease, but this might not hold true for the later phases of the disease, when the therapeutic index becomes narrower. Then the generic with a higher pharmacokinetic exposure (i.e. Cmax and AUC ratios that are higher than 100%), but still within the limits of bioequivalence, may not be able to guarantee the same therapeutic safety of the reference product [15–17].

The efficacy of a medicinal product is affected by several factors, including interactions with other medicines and genetic variability. In the case of clopidogrel, genetic variations of the CYP2C19 enzyme or concomitant use of drugs that inhibit this enzyme, such as proton pump inhibitors [18], introduce variability in the efficacy of clopidogrel. This became known several years after the placement of both products on the market.

A concern on the clinical effectiveness and safety of a generic clopidogrel might arise if the confidence interval is close to the lower acceptance limit. Obviously, this concern would be even more marked for a highly variable drug. Clopidogrel is not considered a highly variable drug; however, an intra-subject variability of 30% has been published in European public assessment reports [18]. The EU’s bioequivalence guideline [3] offers the possibility to widen the confidence intervals for highly variable drugs. While this is understandable and justified from a pharmacokinetic point of view, it could hypothetically in some cases raise additional issues of clinical effectiveness / safety. This is because of the possibility that drug-drug interactions which have not yet been identified or if the product is affected by genetic variability modifying the product’s clinical efficacy.

A challenge during the approval process of generics is when Risk Minimization Measures (RMMs e.g. patient alert cards and educational materials) have been introduced by the brand leader on a voluntary basis, but the safety concerns of the reference product are briefly mentioned in its labelling without contraindications or specific warnings. In this scenario, a generic product can contribute to building the safety profile of the medicine by increasing patient exposure. However, it may not be considered appropriate to set legally binding obligations in the marketing authorization and to impose RMMs on a generic when the reference product lacks these legal obligations [20, 21].

The approval of a generic product containing two strengths that make reference to different reference products (Zometa® and Aclasta® (both contain zolendronic acid)), within the same global marketing authorization, could also be challenging. This is because both reference products have different educational materials highlighting important identified risks, one dealing with the osteonecrosis of the jaw (Zometa®), and the other for renal dysfunction (Aclasta®). However, the educational materials implemented for Zometa® are voluntary, while those for Aclasta® are obligatory. It should be pointed out that the risks of Zometa® and Aclasta® are not the same (since the target patient populations differ) and there are differences in the labelling of both products. Thus, the assessment of the generic medicinal product should be able to harmonise the product information, but neither undermine the results of the evaluation of the reference product, nor disregard the important identified risks and the respective educational materials [22, 23].

### Inspection and Enforcement Challenges

EU regulators rely on the data and analysis of the results from clinical trials which were carried out by applicants in order to reach their opinions / recommendations on the approval / authorisation of medicines. It implies, therefore, that there is an element of trust between regulators and pharmaceutical companies. The system is based on trust. It is known that authorities have finite inspectional resources and they cannot inspect all the parties that are involved in conducting or overseeing research. It is of great concern when questions about the validity of the data in the dossiers are raised, because it gives the perception that fabrication of data is easy [24]. Thus, so much depends on a good inspection system for clinical practices (GCP) as well as for manufacturing practices (GMP). The remit of the EMA’s GCP Inspectors Working Group focuses on the harmonisation and coordination of GCP-related activities at a community level. Bilaterally, the EMA and FDA have signed an agreement to join forces to manage the finite resources, but this has not extended yet to carrying out joint inspections [25]. Internationally, the World Health Organization Prequalification of Medicines Programme (WHO-PQ) coordinates the evaluation and inspection activities of submitted finished pharmaceutical products for pre-qualification. The inspections are performed by a team of inspectors consisting of experts appointed by the WHO (preferably from drug regulatory authorities’ inspectorates, who act as temporary advisers to the WHO) and WHO staff members [26].

## Discussion

One potential solution to the above-described scenarios would be to establish individual bioequivalence studies including long-term efficacy (when applicable and only for certain drugs such as immunosuppressants) and safety follow-ups, rather than average bioequivalence. Post-authorisation, concerns regarding the safety in combination with the efficacy of a medicine, might be raised when a medicine is substituted with another (either “me too” or generic). This is because such concerns are related to the interchangeability of a medicine in a patient in treatment. Individual bioequivalence could take into account variances (intra-subject as well as formulation variances and their effects in subjects) as an important criterion potentially affecting clinical efficacy, when a generic is substituted for a reference product. However, individual bioequivalence remains a theoretical concept which has not yet been proven in clinical practice. In addition, the approval of marketing authorizations submitted with support from individual bioequivalence data has not been implemented. The statistical approach in individual bioequivalence is another important issue, as major efficacy and safety concerns arise due to the possible recognition of wide acceptance ranges.

Another potential solution for certain cases of medicinal products could be to tighten the acceptance range (possibly through the tightening of only one side of the acceptance limits) and to establish therapeutic equivalence. However, there could be limitations of such a proposal (e.g. an asymmetric acceptance range of 90–125%) in clinical practice, as issues of interchangeability within the same patient could be of concern. Switches from the brand leader to the generic, vice-versa, or generic-to-generic occur in many clinical practice settings throughout the world and are increasingly endorsed by governments and third party payers. Therefore, there is a possibility that the acceptance limits for effectiveness would then become larger going from one product to another. In epilepsy, in a scenario where the generic’s confidence intervals during approval were closer to the 80% limit, when a patient is switched from a generic to the brand leader, a greater risk for side effects could be created than the vice-versa case of switching from brand-leader to the generic. Thus, patients could have higher drug plasma concentrations when switched to the originator/brand leader product. Unless justified CIs of 80–125% are accepted by the regulators, narrowing the confidence intervals (both sides) would resolve efficacy and safety concerns in the above scenarios as well as when there are documented drug-induced interactions or genetic variability that decrease a clinical response. It should be noted, however, that such issues have been foreseen in the guidelines and appropriate reference is made to narrow therapeutic index or narrow therapeutic range products. There may be a need to expand these narrow CI limits for additional categories of medicinal products (e.g. products that have had the black symbol and have been under additional monitoring).

Regulators face the challenge to impose RMMs on both brand leaders and generic products when their respective companies may have a different economic interest in the marketing of the products at stake. Originally, the company of the brand leader was the “owner” of the on-patent product, synergizing both responsibilities of risk minimization and economic benefits. After the generic switch, ‘ownership’ and economic interests became fragmented among multiple stakeholders. Recent EU regulations allow for the requirement to implement RMMs as part of the license and to set conditions to monitor the variability of the clinical response of licensed products, both reference products and generics. Generic manufacturers are in a position to be able to set and justify bioequivalence acceptance ranges, but is all the information publicly available to carry this out? Publishing assessment reports by regulators helps, while EU summaries of risk management plans will provide additional information. When in doubt, manufacturers could request scientific advice on their clinical development program.

**Fig. 1.**
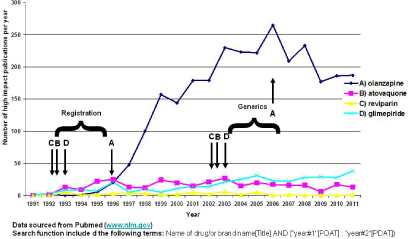
Knowledge generation over time

The EU has started issuing specific monographs for bioequivalence (similar to the FDA), and acceptance ranges are specified there (refer to www.ema.europa.eu). With such an approach, EU regulators must consider what would be the best time to prepare such monographs. Is it just after the originators’ licensure or after some years of clinical experience? Based on the data obtained (Fig. 1), we identify the period upon which licensure of the originator is apt since the clinical data supporting efficacy have been recently generated. Following that, an update to such monographs will be probably required two to three years before coming “off patent”, since this is expected to be on the crest of the knowledge base generated for the specific medicinal product.

Despite recent unfortunate issues with generic drug companies that have raised questions about quality and supply, it is our opinion that few healthcare systems have shunned the generics products altogether. With stepped-up international scrutiny of the industry, national healthcare systems continue to show confidence in generics as cost-effective treatment options.

## Conclusion

This paper discusses broad, but important regulatory issues and challenges faced by regulators during the approval of generic medicinal products that could potentially affect the efficacy and safety of generic medicinal products, since the risk of licensing inappropriate generics may lead to public health consequences and such consequences cannot be withstood by the system. As a result, based on our personal experiences, we have discussed examples and the possibility whether partially changing the rules is appropriate. It should be noted that in the vast majority of instances, the current rules (safety and efficacy) are appropriate, but acceptance limits might need to be tightened for certain medicinal products, and not only due to the active substances’ narrow therapeutic index. Furthermore, we highlight specific scenarios with a substantial likelihood of occurring that could give rise to safety concerns encountered during the approval process of generic applications. Manufacturers should keep these aspects in mind when submitting a marketing authorization application. Similarly, regulators during the assessment process are expected to approve a generic medicinal product, which in the above scenarios could not always be through an easy, clear-cut decision-making process.

## Authors’ Statement

### Disclaimer

The views expressed in this article are the personal views of the authors and may not be used or quoted as being made on behalf of, or reflecting the position of, any national competent authority, the EMA, or one of its committees or working parties or any University.
